# Review of the Biomolecular Modification of the Metal-Organ-Framework

**DOI:** 10.3389/fchem.2020.00642

**Published:** 2020-07-28

**Authors:** Qiqi Xing, Yixiao Pan, Yihe Hu, Long Wang

**Affiliations:** Department of Orthopedics, Xiangya Hospital, Central South University, Changsha, China

**Keywords:** MOF, biomolecular, modification, delivery, nano material

## Abstract

Metal-organ frameworks (MOFs), as a kind of novel artificial material, have been widely studied in the field of chemistry. MOFs are capable of high loading capacities, controlled release, plasticity, and biosafety because of their porous structure and have been gradually functionalized as a drug carrier. Recently, a completely new strategy of combining biomolecules, such as oligonucleotides, polypeptides, and nucleic acids, with MOF nanoparticles was proposed. The synthetic bio-MOFs conferred strong protection and endowed the MOFs with particular biological functions. Biomolecular modification of MOFs to form bridges for communication between different subjects has received increased attention. This review will focus on bio-MOFs modification methods and discuss the advantages, applications, prospects, and challenges of using MOFs in the field of biomolecule delivery.

## Background

The metal-organ framework (MOF), which is also known as a porous coordination polymer (PCP), is a kind of novel nanomaterial composed of metals or metal clusters, chains or layers formed by non-toxic metals (Fe, Zn, Ca, Mg, etc.) and organic compounds, such as carboxylic acid and phosphonic acid (Férey, 2008). PCPs have been widely used in the fields of chemistry and material science in applications such as gas separation and storage (Eddaoudi et al., 2002), sensing (Xu et al., 2011), catalysis (Zhuang et al., 2015), and chromatography (Li et al., 2009; Xiao et al., 2009). MOFs have a porous structure with pore sizes between 0.4 and 6 nm (Zhou et al., 2012). The inherent pore size and structure can be modulated according to the organic-metal compound category and the spatial arrangement. These characteristics enable MOFs to possess open architectures that allow them to combine with, penetrate, and encapsulate a variety of molecules. Therefore, MOFs are ideal materials for storage, protection (Liang et al., 2016b) and carrier functions (Morris and Wheatley, 2008). Recently, non-toxic and biocompatible MOFs, as newly developed materials, have been recommended for use in biological field applications, such as bio-imaging, drug delivery, and electrocatalysis (Huxford et al., 2010; Cai et al., 2015), and these functions were primarily achieved by incorporation of drugs or biological molecules with MOFs (Wang et al., 2015). Increasing numbers of drugs have been combined with different kinds of MOFs, including isoniazid (INH), which is a traditional antitubercular agent that has been loaded onto Fe-MIL-101-NH_2_ nanoparticles to control the release of the drug and improve absorption by macrophages (Wyszogrodzka-Gaweł et al., 2019). An antibiotic (vancomycin) and targeting ligand (folic acid) could also be combined with zeolitic imidazolate framework (ZIF-8) nano MOFs to be delivered to *Staphylococcus aureus* to control infection (Chowdhuri et al., 2017). Furthermore, loading with antitumoral and retroviral drugs to fight cancer and AIDS has greatly inspired MOF research (Horcajada et al., 2009). These successes have promoted the development of MOFs for use in the field of biology.

Biomolecules are special organic molecules produced by living systems, including nucleic acids, proteins, polysaccharides, and lipids, which cannot easily pass through a cell membrane due to their large weight and volume. Biomolecules can also be present on the cell membrane alone, but this occurs with difficulty (Whitehead et al., 2009) owing to the effect of serum nuclease (Keles et al., 2016). Delivery systems have emerged to offer superior thermal and chemical protection for biomacromolecular cargo, which is mainly divided into virus and non-viral vectors (Yoo et al., 2011; Kotterman et al., 2015). Compared with non-viral vectors, the low selectivity, potentially unsafe nature, and insertion mutations (Thomas et al., 2003; Waehler et al., 2007; Mintzer and Simanek, 2009) of viruses have limited their application in living bodies. Non-viral vectors, including organic carriers (liposomes, polymers, peptides, etc.) and inorganic carriers (silica, carbon tubes, calcium phosphate, etc.) (Sokolova and Epple, 2008; Nam et al., 2009), are also considered to be defective (Yin et al., 2014; Chira et al., 2015). Among them, poly-L-lysine, which is a cationic polymer, is one of the most common polymers used for DNA delivery (Khalil et al., 2006; Cheng and Lee, 2016). However, its biotoxicity, as well as its ATPase reduction, autophagy (Lv et al., 2006) and immunogenic responses, cannot be ignored (Tseng et al., 2009; Wan et al., 2013). Additionally, as the only non-viral delivery systems currently in clinical trials, lipid microsystems (Ginn et al., 2018) have been suggested to form large aggregates that hinder the further transportation of nanoparticles in blood (Dash et al., 1999; Das et al., 2015). It is necessary to develop a new biomacromolecule carrier with good biocompatibility and biodegradability.

As porous nanomaterials, MOFs are capable of high loading capacities, controlled release, plasticity, and biosafety, which facilitate the selective transportation of nanomaterials through the porous network (An et al., 2012). MOFs have been proposed as an attractive alternative to mitigate drawbacks that other drug delivery systems (DDSs) face (Abanades Lazaro et al., 2020). Thus, MOFs have already served as a successful drug delivery platform (Zhao et al., 2011). However, there are many differences between drug and biomolecule delivery systems (Zhuang et al., 2017). First, the electrostatic interactions and covalent bonds used to incorporate drugs might not be suitable for the incorporation of biomolecules and MOFs. Second, MOF stability should be a consideration because the addition of biomolecules might change the crystal structure. Third, targeting will be expected. Last, but not the least, a controlled-release effect is another characteristic to be encouraged. Among these requirements, the most important is the effective protection of biomolecules from external factors. In addition, the simplicity and reproducibility of material manufacturing should also be taken into account (Morris and Wheatley, 2008; Imaz et al., 2011). At present, there are two ways to combine MOFs with biomolecules. One method involves growing or depositing metal ions and organics onto a two-dimensional plane to form MOFs films, which are used to physically wrap the target molecule (Horcajada et al., 2011; Shekhah et al., 2011). The other method involves chemical bonding, including surface modification and internal encapsulation (Doonan et al., 2017), which is particularly useful due to its high stability, capacity, and affinity.

This review will focus on the reported methods of bio-MOFs functional synthesis, including surface modification and internal encapsulation, and further discuss the advantages, applications, prospects, and challenges of MOFs in the field of biomolecule delivery.

## MOF Biofunctionalization by Surface Modification

**Figure 1:**
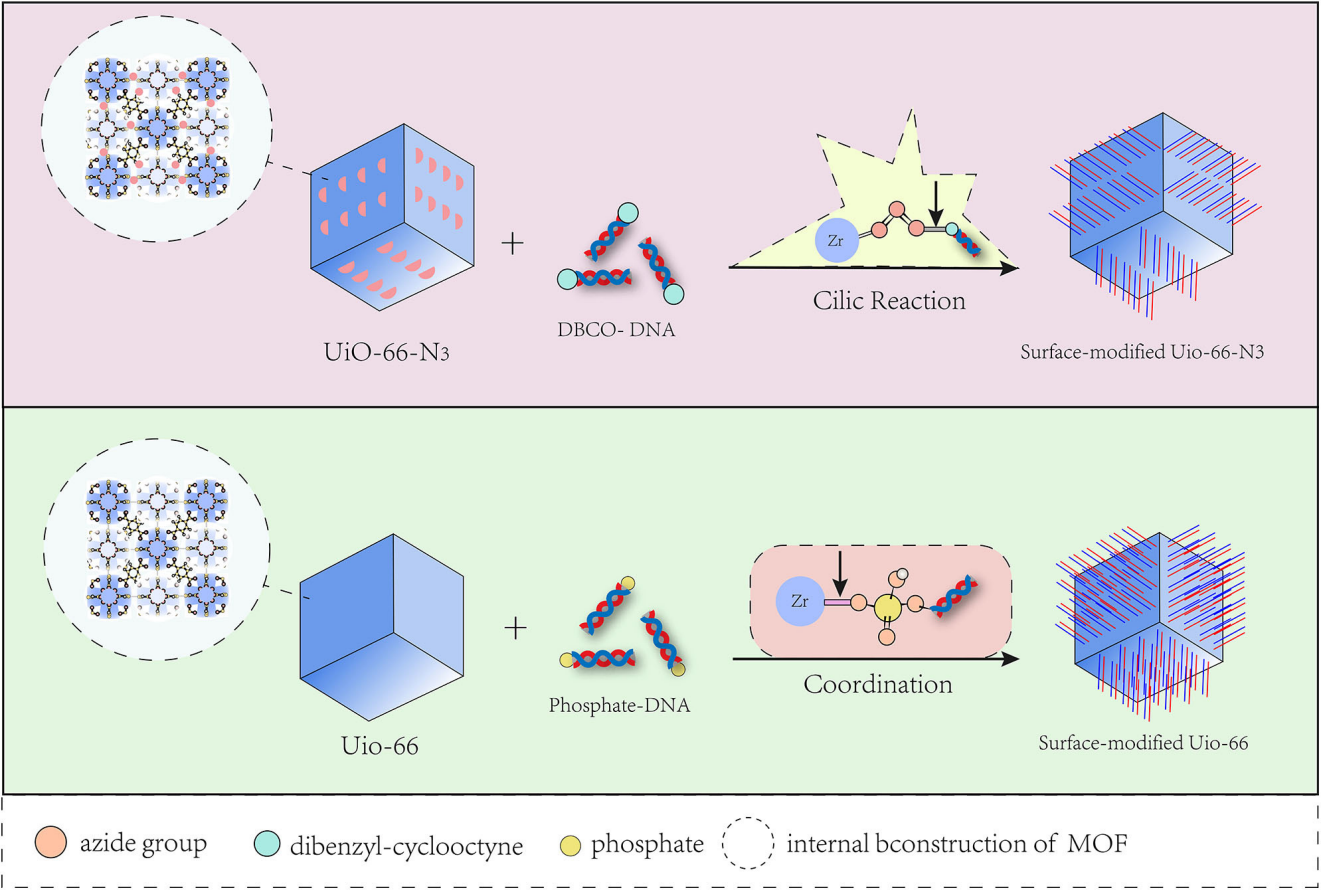
Two methods for surface modification to MOF by DNA.

In pioneering research, the biochemical surface modification of nanomaterials has been applied to biological probes and cell imaging (Wang D. et al., 2019). To date, MOFs have been modified on their surface to meet specific requirements and achieve biological functionalization, such as targeted delivery and localized release (Meng et al., 2018). These characteristics greatly improve the performance and bioutilization of MOFs (Cai et al., 2017; Huang et al., 2018). Consequently, there are two generalizable methods that postsynthetically functionalize the bulk MOF structure with biomolecules (Wang et al., 2015). The first method is the covalent combination of the modifier with an anchor on the surface of prepared MOFs before MOF synthesis. Unlike the modification of a pre-synthesized organic linker, the second method involves the coordination of the modifier directly on the surface of the post-synthesized MOF. The chelation of metal ions with target molecules is responsible for the connection (Deria et al., 2015). The unsaturated coordination metal sites on MOFs allow for the incorporation of biomolecules, and UiO-66 will be used to discern the two methods (Figure 1).

Via the first method, the conjugation of nucleic acid and azide-functionalized UiO-66-N_3_ (Zr_6_O_4_OH_4_(C_8_H_3_O_4_-N_3_)), which is transferred from UiO-66, was produced (Morris et al., 2014). Alkane terminal ligands could react with the azide group in UiO-66-N_3_ via click reactions, which have been utilized to interface MOFs in bulk with a variety of organic functionalities. By the click reaction, UiO-66-N_3_ was able to interact with dibenzyl-cyclooctyne (DBCO)-functionalized DNA and further realize cellular entry. Furthermore, the functionalized MOFs gained the ability to hybridize with diverse complementary nucleic acids in a sequence-specific fashion, which provided the possibility of the widespread bioapplication of this kind of MOF. However, the degree of DNA surface combination obtained by the first approach was approximately two times lower than that achieved by the other method, in which the surface modification of oligonucleotides resulted from metal–phosphate coordination through modified DNA and unsaturated metal sites on the MOF surface (Wang S. et al., 2017). In that process, chemically modified phosphonamidites at either the 3′ or 5′ end of the oligonucleotide were added into the colloidal suspension of synthesized UiO-66, and then the terminal phosphate moiety was adsorbed onto the MOF nanoparticles by coordination with the solvent-bound Zr sites (Deria et al., 2015). The affinity of the terminal phosphate for the Zr centers was better than that of the internal phosphodiester bonds of oligonucleotides because of the increased steric hindrance encountered by the internal phosphodiester. In addition, the DNA surface coverage directly correlated with the surface second building unit (SBU) density, metal–phosphate coordination number, and bond strength of the MOF. Therefore, increased SBU coordination numbers yield higher DNA functionalization densities. Additionally, it should be mentioned that this approach to DNA-modified MOFs was independent of the category of organic linkers and was broadly applicable to a variety of metal clusters. This means that it is a general way to synthesize the regular structure of surface-modified DNA-MOFs. Based on this method, a research team then designed a nucleic acid-MOF nanoparticle using NU-1000 and PCN-222/MOF-545, which acted as a host to easily and effectively deliver a variety of proteins into cells (Wang S. et al., 2019).

Through in-depth research, many kinds of biomolecules have been developed for the surface modification with different MOFs, such as oligopeptides that form metal-peptide frameworks with copper and calcium due to carboxylic acid chelation (Mantion et al., 2008). Surface combination has become an important and general way to obtain biofunctionalized MOFs.

## MOF Biofunctionalization by Internal Modification

**Figure 2:**
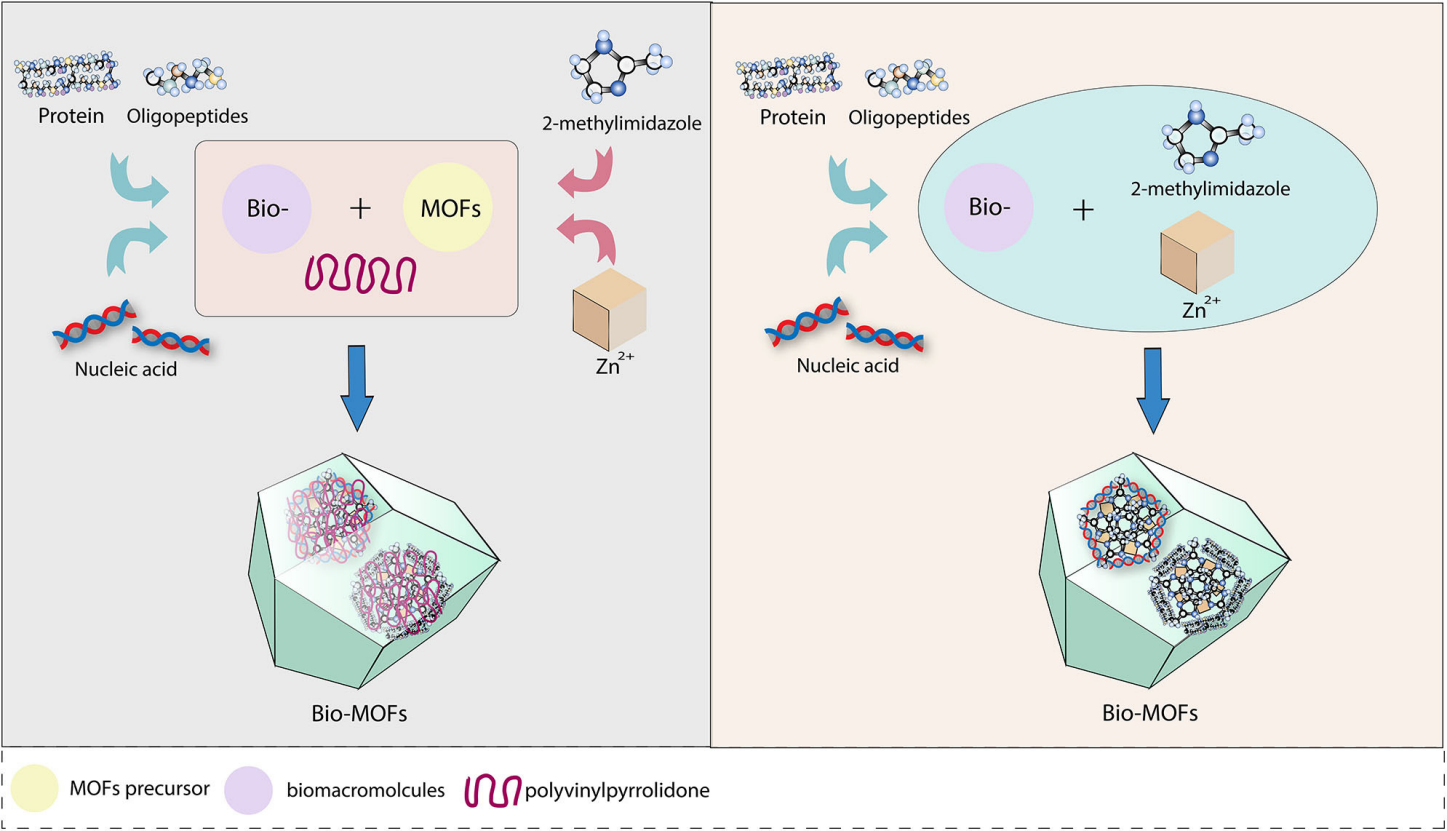
Two methods for internal modification to MOF by biomolecule.

Unlike surface modification, internal modification involves a higher-level combination, which involves the encapsulation of molecules by MOFs. The molecules can be better protected and bound more tightly by internal modification than by surface modification. Through encapsulation, MOFs could act as carriers to deliver large drugs and biomolecules, as well as to achieve the condition-triggered release of drugs from a vehicle (Roth et al., 2018). Compared with drugs, proteins, and nucleotides have more complicated multistructures, which means that conformational changes are always unavoidable and might disrupt their function (Wickner, 2005) when they are transported through nano channels to enter the cell. Therefore, a carrier would be necessary and important. Among nano delivery systems, MOFs are novel and outstanding. Taking enzymes as an example, MOF (Hudson et al., 2008) crystals have an increased encapsulation efficiency, recyclability, and an excellent enzyme-catalytic performance (Rapoport, 2007) compared to mesoporous silica (Lykourinou et al., 2011). However, biomacromolecules similar in size to MOF pores were hardly able to be loaded into the framework via postsynthetic infiltration (Chen et al., 2012). Recently, two methods for encapsulating target molecules into MOFs at the beginning of synthesis were proposed. Here, we utilize zeolitic imidazolate frameworks (ZIFs) as an example because of their growth characteristics under mild biocompatible conditions (Zhuang et al., 2014) to describe two internally modified methods (Figure 2), which are coprecipitation (Lu et al., 2012; Shieh et al., 2015) and biomimetic mineralization (Liang et al., 2015).

The coprecipitation method was proposed by Lyu et al. (2014), who added a solution containing cytochrome c (cyt c) and polyvinylpyrrolidone (PVP) to a methanol solution of 2-methylimidazole and zinc nitrate hexahydrate to achieve cyt-ZIF nanoparticles. A transmission electron microscope (TEM) showed that Zn^2+^ and 2-methylimidazole were first assembled into rod-shaped crystals. Then, rhombohedral dodecahedral crystals formed 24 h later, indicating that ZIF-8 was formed first, before the protein was embedded. In addition, Liu demonstrated the generality of coprecipitation by loading horseradish peroxidase and lipase onto ZIF-8 and ZIF-10. The biomimetic mineralization method was inspired by a natural process according to the specific ability of amino acids, peptides, and enzymes to concentrate inorganic cations in biominerals (Trzaskowski et al., 2007; Hwang et al., 2013). This is a new and simple method to rapidly encapsulate proteins, enzymes, and DNA into MOFs, as suggested by Liang et al. (2015). Bio-ZIF could be prepared by mixing an aqueous solution containing 2-methylimidazole and bovine serum albumin (BSA) with an aqueous solution of zinc acetate at room temperature, depending on the nucleation of the ZIF-8 precursor. Coordination between the Zn cations and the carbonyl group of the proteins is formed naturally. Different from the crystals separated from the mixture of ZIF-8 and BSA, the BSA-ZIF synthesized by mineralization was shown to encapsulate BSA and perfectly retain the structure of the enzyme. When the biomimetically mineralized ZIF-8 layer was removed via pH modulation, the released biomacromolecule retained its native activity. Additionally, the category of encapsulated biomacromolecules was enlarged by biomimetic mineralization, and the crystal morphology of bio-MOF had a unique dependence on the biomacromolecule.

Coprecipitation and biomimetic mineralization are generally known as “one-pot” methods because in both methods, biomolecules are initially mixed with MOF precursors and surrounded by grown MOF materials instead of occupying cavities. The difference is that PVP is utilized to functionalize the precursor and optimize the crystallization of ZIF-8 in coprecipitation. PVP is an amphiphilic, non-ionic polymer used as an efficient MOF nucleating agent to support size and shape control and stabilize nanoparticles during synthesis (Li and Zhang, 2006). In the mineralization method, the nucleation of precursors in an aqueous solution allows ZIF-8 to form a protective layer without PVP. According to a comparative study of the two methods, the loading rate of protein was equivalent. Compared with that of free enzymes, biomimetic mineralization expanded the temperature range of enzymatic biological activity, that is, within a certain temperature range, biomimetic mineralization would have a better protective effect on the enzyme than coprecipitation (Liang et al., 2016a). In addition, the stability of the enzymes encapsulated via biomimetic mineralization was enhanced, probably due to the rigid ZIF-8 structure restricting the structural rearrangement at elevated temperatures (Hartmann and Kostrov, 2013). Moreover, the distribution of cavities throughout the crystals was analyzed by thermal enzymatic decomposition, which indicated that this was more likely to expose the enzyme to the external environment in coprecipitation. In summary, mineralization was the preferred choice for biomolecule encapsulation (He et al., 2016).

In addition, the colloidal stability of the synthetic material should be considered seriously in terms of physiological factors, for example, protein-containing solution. The surface coating is proposed to stabilize the structure of nanoparticles, such as silica, (Della Rocca et al., 2011) hydrophobic polydimethysiloxane (PDMS) (Zhang et al., 2014), and hydrogel. Hydrogels are structurally various and functional materials composed of cross-linked hydrophilic polymers. The network endows a hydrogel with stability, which is a prominent advantage in embedding and protecting other materials. Additionally, the local release of cargo from a hydrogel can be achieved by degradation under specific environmental conditions. For instance, Cu-MOF has been embedded in poly-(polyethyleneglycol citrate-co-N-isopropylacrylamide) (PPCN), which is an intrinsically antioxidant thermoresponsive citrate-based hydrogel, and MOF NPs didn't degrade in a protein solution as a result of protection by the hydrogel coating (Xiao et al., 2016). The pH-sensitive carboxymethylcellulose (CMC) biopolymer was used to protect and carry the 5-FU encapsulated MOF-5 nanohybrid (5-FU@MOF-5) through the digestive system (Javanbakht et al., 2019). It should be mentioned that high biocompatibility and biodegradability are crucial properties of hydrogels, which bring numerous possibilities for use in the biomedical field (Fu et al., 2018). However, electronic interactions between positively charged metal ions in MOFs and negatively charged ions in hydrogels may limit gelation. The type of metal atoms in the MOF must be considered. In a word, the hydrogel coating method can be applied to entrap MOF NPs as a mechanism to slow degradation and thus prevent aggregation.

## Advantages, Prospects, and Challenges

### Bio-MOF Advantages

#### MOFs Remarkably Protect the Activity of Biomolecules

**Figure 3:**
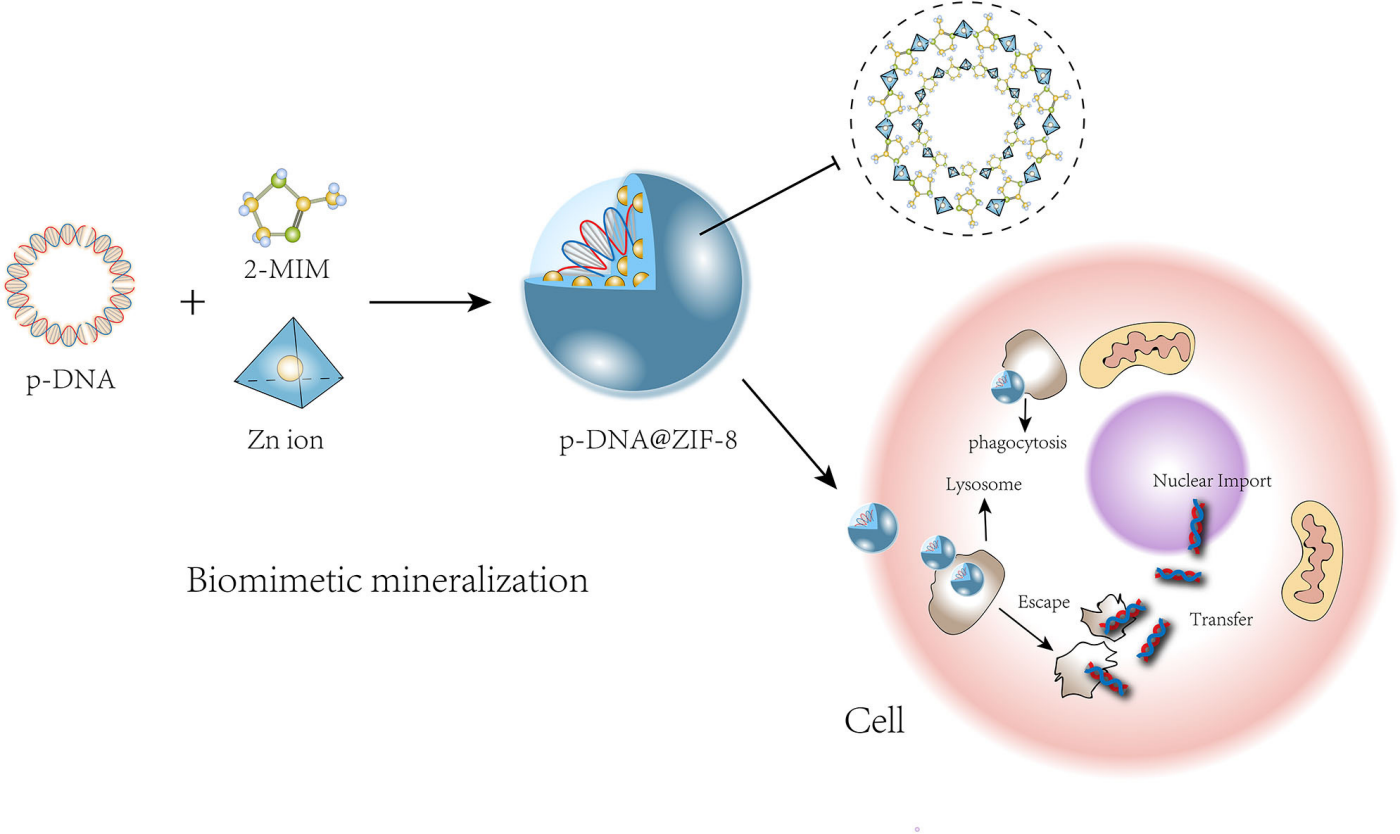
Process of pDNA growing with ZIF-8 precursors and transportion into cell.

As nanoparticles synthesized from organic and inorganic components, MOFs are able to combine with a variety of molecules and offer superior thermal and chemical protection for their cargo (Liang et al., 2015). MOFs have been successfully developed not only in the field of drug release (Zhuang et al., 2014) but also in the study of biomacromolecules. The “micro enzyme” catalase (MP-11) and high-molecular weight molecules such as cytochrome c (104 amino acids), organophosphorus acid anhydrolase (440 amino acids) (Li et al., 2016), and bacillus subtilis lipase (BSL2) could be encapsulated by MOFs (He et al., 2015). The framework could effectively maintain the skeletal integrity, biological activity, and reusability of proteins (Cao et al., 2016). It was reported that two enzymes, α-glucosidase (GAA) and glucose oxidase (GOx), could be assembled on one Cu-MOF molecule to generate a bifunctional hybrid enzyme-catalytic framework reactor, which could be utilized for the highly sensitive and stable screening of GAA inhibitors (Zhong et al., 2019). In terms of nucleic acids, low-molecular weight (MW) molecules such as oligonucleotide CpG ODNs (Zhang et al., 2017) and ssDNA (11, 22, 33, and 53 nucleotides) (Peng et al., 2018) have been wrapped and combined with MOFs. However, to the best of our knowledge, it is challenging to safely deliver nucleic acids with a high MW. The latest research showed that even macromolecular plasmid DNA could be successfully embedded into a ZIF-8 carrier by the biomimetic mineralization method (Li Y. et al., 2019), so that the pDNA could be protected when it passed through the cell membrane into the lysosome and to then be released around the cell nucleus (Figure 3). Additionally, a 25-kD polyethyleneimine (PEI) capping agent was added to improve the ZIF-8 crystal structure strength, loading capacity, pH-responsive release, and binding affinity to pDNA. The synthetic nanoparticle pDNA@ZIF-8-PEI 25 kD performed better than pDNA@ZIF-8, with improved gene expression and high transfection efficacy in various types of cells, possibly because of the enhanced positive charge that facilitated binding and internalization of the nucleic acid molecule. In summary, MOFs can be used as carriers of various biomolecules and play an effective protective role during delivery.

#### New Characteristics of MOFs Endowed by Molecules

##### Stability

MOF nanoparticles can be structurally affected by biomolecular modification. For example, the insertion of an enzyme was beneficial to the construction process of MOFs (Liang et al., 2016a). The binding of oligopeptides could lead to adaptive changes in metal-peptide frameworks (MPFs), which showed differences in porosity and organometallic framework strength according to the different types of amino acids used, such as glycine, threonine, and alanine (Carlos et al., 2012). In addition, lipid-functionalized MOFs have shown better stability in aqueous solution than unmodified MOFs (Wuttke et al., 2015; Zhuang et al., 2015). Liu et al. (2018) proved that Zr MOF nanoparticles bound to single-stranded DNA (ssDNA) showed better stability than did unbound particles over 24 h. In addition, the interaction strength and pore geometry influence the absorption and release kinetics, as well as the way the matrix diffuses inside and outside the pores (Carlos et al., 2012).

##### Targeting Movement and Local Release

Modification not only changes the MOF structure but also endows new characteristics. Cell-targeting capabilities should be mentioned first. The mechanism of targeting is always related to the bio-MOF's bioactive group. The most common targeting method is the coupling of the ligand-receptor through distinguishing the specific recipient from the other cells. A manganese MOF modified by small cyclic arginine-glycine-aspartate (RGD) peptides has been verified to target angiogenic cancer cells well by binding to the upregulated αvβ3 integrin (Taylor et al., 2008). A terminal cyclic RGD-SH peptide was modified onto a hybrid MOF, and the nanoparticle was proven to have a targeting ability for HeLa cells (Wang D. et al., 2018). Folic acid-bovine serum albumin (FA-BSA) was a significant targeting connector for cancer cells, which resulted from efficient internalization via FA-receptors-mediated endocytosis (Jiang et al., 2018). Polysaccharide hyaluronic acid (HA) acts as a cancer-targeting ligand when connected with MOF, too, because it recognizes the overexpressed CD44 that occurs in many cancer cells (Liu et al., 2016; Kim et al., 2019). Another important mechanism of bio-MOFs' targeting movement is the sensing of chemical gradients in environments, which has previously been discovered in bacteria. We can achieve directional motion by endowing MOFs with pH-sensitive biomolecules. Ikezoe et al. (2015) encapsulated the diphenylalanine (DPA) peptide, which could facilitate Cu-MOF movement based on an asymmetric surface tension distribution through the dissolution and self-assembling character of DPA. The solubility of PDA is sensitive to pH gradients. With a higher pH, the solubility of the DPA peptide increased, leading the MOF to lose the surface gradient, thus terminating the motion. In this way, the directional movement of MOF NPs was achieved.

In addition, controllable release is another exciting characteristic (McKinlay et al., 2010), which benefits from the fact that some kinds of MOFs can be degraded in certain environments (Park et al., 2006; Lin and Anseth, 2008). ZIF-8 has good biodegradability under acidic conditions (Della Rocca et al., 2011; Zheng et al., 2016). The ZIF-8/CpG-ODN complex showed good stability in a physiological environment, but the complex effectively released CpG ODNs under acidic conditions corresponding to the endolysosome identified by Toll-like receptor 9 (TLR 9). Moreover, ZIF-8 could significantly improve the uptake of CpG ODNs by RAW264.7 cells and further promote the secretion of immune cytokines *in vitro* and *in vivo* (Li Y. et al., 2019).

### Applications, Prospects, and Challenges

As a novel platform for bioapplications, MOFs have made rapid progress and have constantly provided new methods for biomolecular delivery systems. MOF formation occurs because of the coordination between metal atoms and the organism, and different numbers and categories of metals or organic compounds will produce a variety of constructs, such as those resulting from bimetallic organism synthesis. Recently, a new multivariate modulation of Zr-MOF UiO-66 (Abanades Lazaro et al., 2020) was reported. Multivariate modulation allows the incorporation of up to three drugs containing either carboxylates or phosphates as metal-binding units to coordinate with the defect sites of metal clusters in UiO-66. The one-pot synthesis of solvothermal compounds retained Uio-66 crystallinity and porosity so that other drugs could be further loaded. This research revealed the broader prospects and bioapplications of MOFs. MOFs are also used in the field of bioprobes. The Cu-MOF was combined with a ssDNA probe labeled with carboxyfluorescein through electrostatic interactions and/or hydrogen bonding, which was used to detect Hg via the coordination motif between Hg and ssDNA (Huang et al., 2019). Another new application involved encapsulating viral nanoparticles to produce a vaccine carrier, so that the integrity of the virus and the biosafety and immunogenicity of the overall composite was enhanced due to the non-toxicity and good biocompatibility of the MOF (Luzuriaga et al., 2019).

However, the use of MOFs as potential carriers for the intracellular transmission of proteins (Liang et al., 2015) and nucleic acids (Wang S. et al., 2017; Wang Z. et al., 2017; Peng et al., 2018) is still in the preliminary stage (Alsaiari et al., 2017; Chen et al., 2018). There are some barriers that need to be resolved in the process of the bioapplication of MOFs. For example, in the future, the induction and growth-affecting factors of proteins or nucleic acids in morphological structures need further study, which will allow large double-stranded DNA molecules to be wrapped in MOFs. The biological stability of bio-MOFs is another constraint. Coating may be a good choice to escape from immune system, such as poly-lactide-co-glycolide (PLGA) and cell membrane (Wang L. et al., 2018; Li J.-Y. et al., 2019). We team has invented a cell membrane-coated nanodrug deliver system to improve biocompatibility, and it has been proved the embedded drug can cleverly escape identification and clearance from the immune system, effectively prolong the blood circulation time and accurately accumulate in the target tumor tissues (Wang et al., 2020). In addition, some researchers have cautioned that most enzyme-MOF research has been focused on enzyme encapsulation in particle form, which means that solid support is essential, but this may limit the material flexibility for further practical applications (Izzah Binti Mohammad et al., 2018). Therefore, the use of versatile modalities, such as flexible ZIF-8 thin films, for the synthesis of bio-MOFs is required.

Currently, bio-MOF cascade reactions in cells have been put forward as a novel strategy. How to utilize the synergy of modified molecules in nanoparticles during working processes in organisms is still an unanswered question. For instance, in multimodal cancer therapies, the synergistic cancer starvation/ROS-mediated/chemotherapy strategy has been designed to cleverly work together in cancer cells. Glucose oxidase (GOX) modified onto the surface of MOF(Fe) catalyzes glucose into hydrogen peroxide (H_2_O_2_) and gluconic acid (H^+^) in cancer cells, and then H^+^ can degrade the organic framework to release camptothecin (CPT) for chemotherapy (Liu et al., 2019). In my opinion, to date, the research of bio-MOFs has always been focused on single molecules or on multiple molecules acting independently in unrelated processes. This kind of therapeutic effect is slight, but the effects will be different when we make use of all linked and correlated nodes in entire reaction chains, such as the chain of ROS-induced oxidation. In the latest research, MOF(Fe) is used to promote ROS-induced oxidative damage in cancer cells, and chloroquine modification will inhibit lysosome autophagy, so that this nanodrug can cut off the self-protection node under the oxidative stress chain and improve the anti-cancer effect (Yang et al., 2020). As we can see, the regulation of multiple reactive nodes in a biological chain can be achieved by setting the reaction times of substances loaded onto MOFs. I believe that achieving synergistic effects to maximize the efficiency of bio-MOFs will be a point that attracts the focus of scientists.

Finally, the development direction of bio-MOFs must be toward clinical applications, and transformations are in development, such as targeted protein or DNA biosensors (Osman et al., 2019) and delivery (Wang S. et al., 2019). There is a wider range of utilization of bio-MOFs. Through interdisciplinary research, bio-MOFs will have a significant impact on various fields including chemistry, genetics, biology, and materials science (Wu et al., 2018). We hope that genetic diagnosis, biological-targeted therapy, and therapeutic drug encapsulation can be improved through the use of MOFs in the future.

## Author Contributions

QX, YH, and LW contributed conception and design of the study. QX wrote the first draft of the manuscript. LW revised the manuscript. YP wrote sections of the manuscript. All authors contributed to manuscript revision, read, and approved the submitted version.

## Conflict of Interest

The authors declare that the research was conducted in the absence of any commercial or financial relationships that could be construed as a potential conflict of interest.
